# Tractography of the Corpus Callosum in Huntington’s Disease

**DOI:** 10.1371/journal.pone.0073280

**Published:** 2013-09-03

**Authors:** Owen Phillips, Cristina Sanchez-Castaneda, Francesca Elifani, Vittorio Maglione, Alba Di Pardo, Carlo Caltagirone, Ferdinando Squitieri, Umberto Sabatini, Margherita Di Paola

**Affiliations:** 1 Clinical and Behavioural Neurology Department, Istituto di Ricovero e Cura a Carattere Scientifico Santa Lucia Foundation, Rome, Italy; 2 Radiology Department, Istituto di Ricovero e Cura a Carattere Scientifico Santa Lucia Foundation, Rome, Italy; 3 Centre for Neurogenetics and Rare Diseases, Istituto di Ricovero e Cura a Carattere Scientifico Neuromed, Pozzilli, Italy; 4 Neuroscience Department, University of Rome “Tor Vergata,” Rome, Italy; 5 Department of Internal Medicine and Public Health, University of L’Aquila, L’Aquila, Italy; Louisiana State University Health Sciences Center, United States of America

## Abstract

White matter abnormalities have been shown in presymptomatic and symptomatic Huntington’s disease (HD) subjects using Magnetic Resonance Imaging (MRI) and Diffusion Tensor Imaging (DTI) methods. The largest white matter tract, the corpus callosum (CC), has been shown to be particularly vulnerable; however, little work has been done to investigate the regional specificity of tract abnormalities in the CC. Thus, this study examined the major callosal tracts by applying DTI-based tractography. Using TrackVis, a previously defined region of interest tractography method parcellating CC into seven major tracts based on target region was applied to 30 direction DTI data collected from 100 subjects: presymptomatic HD (Pre-HD) subjects (n = 25), HD patients (n = 25) and healthy control subjects (n = 50). Tractography results showed decreased fractional anisotropy (FA) and increased radial diffusivity (RD) across broad regions of the CC in Pre-HD subjects. Similar though more severe deficits were seen in HD patients. In Pre-HD and HD, callosal FA and RD were correlated with Disease Burden/CAG repeat length as well as motor (UHDRSI) and cognitive (URDRS2) assessments. These results add evidence that CC pathways are compromised prior to disease onset with possible demyelination occurring early in the disease and suggest that CAG repeat length is a contributing factor to connectivity deficits. Furthermore, disruption of these callosal pathways potentially contributes to the disturbances of motor and cognitive processing that characterize HD.

## Introduction

Huntington’s disease (HD) is neurodegenerative process caused by CAG repeats in the protein Huntington (HTT) gene [1]. Despite our understanding of HD genetics, the pathogenesis that results in the phenotype remains unclear since disease progression seems to be influenced by factors other than simply the mutation length [2].

The typical HD phenotype is characterized by middle to late age of onset, and by early specific neurodegeneration of striatal neurons. Many studies have documented the deleterious effect HD has on the brain, (for review see: [3]). Furthermore, it has been shown that presymptomatic (Pre-HD) subjects may have brain atrophy up to 10 years before disease manifestation [4], [5]. However, less is known about the effects of HD on white matter (WM) fibers. As opposed to gray matter where a variety of cell bodies (neurons, astrocytes, and oligodendrocytes) and processes (axons, dendrites, and myelin) coexist, WM fibers are composed primarily of myelin and axons. Thus, data on WM modifications in HD can provide the clearest support for the hypothesized process of HD pathogenesis: such as breakdown of early and heavily myelinated fibers and/or axonal degeneration [6].

The corpus callosum (CC) is an ideal WM fiber to investigate. This is because the CC is the largest WM fiber tract in the brain and it contains more 200 million WM fibers that transfer information between the hemispheres. These fibers arise from pyramidal cells that are affected early in HD [7]. The fibers vary in size and age of myelination with larger and early myelinated fibers seen in posterior regions and smaller and late myelinating fibers found in anterior regions [8], [9]. Because of this, the CC has been extensively studied in a number of neurological and psychiatric disorders [10], [11], [12]. Therefore, analyzing CC structure may elucidate the pathological process and regional differences HD has on WM myelination and/or axonal damage. Furthermore, since few callosal fibers arise from cortico-striatal neurons, callosal changes can give a different view of the HD physiopathology.

Diffusion tensor imaging (DTI) is the most frequently magnetic resonance imaging (MRI) technique used to study WM fibers changes. DTI approach is a non-invasive technique, which uses the local water diffusion in the brain tissues to study microstructural aspects of WM anatomy [13]. Though the biological determinants of diffusion parameters are not yet fully understood, (for an in-depth discussion on diffusion imaging see [14]), it is agreed that it is a sensitive modality to microstructural tissue properties. Reductions in fractional anisotropy (FA) could reflect decreased numbers of fibers or indicate reduced axonal myelination [15]. Furthermore, axial diffusivity (AD), a measure of how fast diffusion occurs in the preferred direction, has been shown to be sensitive to the number of axons, as well as their coherence [16]. Increased AD is related to WM axonal atrophy likely associated with Wallerian degeneration although in some cases it may be attributed to increased fiber organization [17]. Radial diffusivity (RD) is a measure of how fast diffusion occurs in the perpendicular direction. Increased RD is thought to reflect reductions in myelination [18], [19], [20].

Recent DTI studies suggest that abnormalities in HD extend to WM tracts. Two such studies showed CC degeneration in HD patients [21], [22] with early degeneration of cortico-cortical connectivity [23] likely contributing to a deficit in associative cortical processing. Two others have suggested that Pre-HD subjects exhibit WM fibers deficits in the CC [24], [25] and motor cortico-striatal circuit connectivity. Furthermore, reduced CC density in the isthmus predicted the “HD development” index [24] and early changes in CC, thalamus, sensorimotor and prefrontal region correlated with clinical function [25]. As such, DTI data may be particularly useful in identifying early and progressive WM changes in HD.

A relatively new step in MRI callosal WM abnormalities investigations is to apply DTI-based tractography, which allows the precise mapping of CC tract anatomy within subjects. The tractography method used in this study has the advantage to not be sensitive to the confounds of voxel-based registrations [26]. Furthermore, it provides regional specificity in anatomical modifications within the CC, by incorporating WM connectivity information. DTI-based tractography, indeed, can map apparent connectivity of voxels in the CC to specific cortical areas, since callosal fibers keep the topology of the orbital–frontal–parietal–occipital lobes.

DTI-based tractography has been reliably used to examine WM in a large number of studies because it allows the investigator to address whether disturbances of WM connectivity occur within particular cortical networks [27], [28], [29], [30]. That is, because the diffusion tensor approximating local diffusion is determined by the local tissue architecture, it can be used to generate an *in vivo* visualization of WM tracts [31], [32], [33], [34]. Although some doubt exists that tractography may generate sufficiently reproducible neuro-anatomical detail for use in quantitative analyses, evidence supports that tracking results agree well with postmortem definitions [35], [36].

To our knowledge, no studies have been published using tractography to investigate regionally specific callosal WM abnormalities in HD and Pre-HD, in spite of the valuable information this approach can provide in terms of callosal WM connectivity modification.

The main aims of our study were 1) to explore the biological mechanism underlying callosal WM changes in a large cohort of subjects (25 HD patients and 25 Pre-HD, and 50 healthy subjects), we predicted that demyelination occurs prior to axonal degeneration in the CC; 2) to individuate a regional specificity in the CC changes, characteristic of the HD pathology, we predicted that the CC and each of the segments would be disturbed in HD with the most severe disturbances located in tracts with target regions in motor areas; 3) to investigate a possible association among genetic, cognitive, motor data and CC fiber values. We predicted that CAG repeat length would be negatively associated with CC microstructure. Lastly, we hypothesized that cognitive and motor scores in patients would be associated with CC fiber organization.

For these purposes, we used both DTI and DTI-based Tractography. We examined the CC in its entirety and segmented into its seven sub regions based on unique target areas: the orbital frontal (OF), anterior frontal (AF), superior frontal (SF), superior parietal (SP), posterior parietal (PP), temporal (Temp), and occipital (Occ). These tracts are easily identifiable using a multiple region of interest (ROI) approach in DTI data [37], [38].

## Materials and Methods

### Ethics Statement

The Santa Lucia Foundation Research Ethics Committee approved this study. All participants had the cognitive capacity to understand the research protocol and gave their oral and written consent. For those participants with disabling motor impairment, their proxies provided signature on the written informed consent. Consent was obtained according to the Declaration of Helsinki and the Santa Lucia Foundation Research Ethics Committee approved the study.

### Subjects

Subject demographics and clinical assessments are outlined in Table 1. HD patients (n = 25) and Pre-HD subjects (n = 25), underwent a genetic test (abnormal CAG repeats ≥36) and were examined clinically by the same neurologist with expertise in HD. All individuals were assessed using the Unified Huntington’s Disease Rating Scale (UHDRS), which includes motor, cognitive, behavioral, and functional subscales [39]. Each section consists of a multistep subscale. The motor section measures eye movements, limb coordination, tongue impersistence and movement disorders (such as rigidity, bradykenisia, dystonia, chorea, and gait disturbances). A higher score means more motor impairment. The cognitive scale mainly evaluates executive function. A higher score means better cognitive performance. The behavioural section investigates the presence of depression, aggressiveness, obsessions/compulsions, delusions/hallucinations and apathy. A higher score means more impairment. The functional assessments include the HD functional capacity scale (HDFCS), the independence scale and a checklist of common daily tasks. All three scales mainly investigate independence in daily life activities. The HDFCS is reported as the total functional capacity (TFC) score (range 0–13) and is the only functional subscale with established psychometric properties (including inter-rater reliability and validity), which are based on radiographic measures of disease progression. Thus, the TFC score is used worldwide to determine patients’ HD stage. On the independence scale, the investigator indicates whether the patient can perform the task that evaluates independence level (range 10–100). The checklist (functional assessment) is summed by giving a score of 1 to all “yes” answers (range 0–25). Pre-HD are defined as those subjects whom the suspected clinical diagnosis is confirmed by DNA analysis, which revealed (CAG)(n) expansion into the range characteristic of Huntington disease (>40 repeats), but who do not have manifested Huntington’s disease symptoms yet defined by a total motor score of <5 in the UHDRS and cognitive and behavioural assessment within the normality. The Disease Burden index, a measure of disease severity, was used according to the already described formula (age×[CAG-35.5]), where CAG is the number of CAG repeats [40]. A higher score reflects increased disease severity. The Mini-Mental State Examination (MMSE) [41], which measures global cognitive functioning, was administered to Pre-HD subjects and HD patients. A lower score reflects greater impairment.

**Table 1:** Sociodemographic and clinical characteristics of patients and control subjects.

	Pre-HD (n = 25)	HD (n = 25)	Controls (n = 25)	Fisher’s Exact Test;F or T Test	df	p
**Characteristics**						
Gender male/female	16/9	14/11	30/20	0.367	2	0.833
Age (years±SD)	37.44±7.01	47.40±14.53	42.88±12.48	4.349	2	0.012^b††^
CAG repetition length	43.28±2.17	46.68±6.80	NA	−2.380	48	0.021^a^
MMSE	27.82±1.24	24.97±3.23	NA	3.682	38	0.001^b^
UHDRS Motor	8.00±9.28	37.22±13.18	NA	−8.695	44	0.001^a^
UHDRS Cognitive	257.80±42.34	142.65±50.35	NA	8.046	41	0.001^b^
UHDRS Behavioral	7.67±7.84	18.39±9.13	NA	−4.160	42	0.001^a^
UHDRS Functional	25±0	17.91±5.68	NA	5.984	44	0.001^b^
TFC	13±0	8.39±2.37	NA	9.329	44	0.001^b^
Independence scale	99.8±1.04	78.04±12.49	NA	8.312	44	0.001^b^
Disease burden	292.3±87.52	458.6±104.75	NA	–6.091	48	0.001^a^

HD = Huntington’s disease; Pre-HD = gene-positive, without motor symptoms; SD = standard deviation; df = degrees of freedom; CAG, trinucleotide repeat number; MMSE = Mini Mental State Evaluation; UHDRS = Unified Huntington’s Disease Rating Scale; TFC = total functional capacity; NA = not available; ^††^ = T-student, Bonferroni correction.

a Pre-HD<HD (when referred to a cognitive scale comparison or CAG repetition, higher punctuations mean greater impairment).

b Pre-HD>HD (when referred to a cognitive scale comparison, higher punctuations mean lesser impairment).

* MMSE: Missing data for 5 Pre-HD subjects & 5 HD patients.

* UHDRS Motor: Missing data for 2 Pre-HD subjects & 2 HD patients.

* UHDRS Cognitive: Missing data for 5 Pre-HD subjects & 2 HD patients.

* UHDRS Behavioral: Missing data for 4 Pre-HD subjects & 2 HD patients.

* UHDRS Functional: Missing data for 2 Pre-HD subjects & 2 HD patients.

* TFC: Missing data for 2 Pre-HD subjects & 2 HD patients.

Fifty individually healthy subjects were recruited from the community. Patients in the advanced stages of disease (Stages III and IV) and/or with traumatic brain injury or focal lesions were excluded.

### MRI Data Acquisition

All MRI data was acquired on a 3 T Allegra MRI system (Siemens, Germany) using a birdcage head coil. Scans were collected in a single session, with the following pulse sequences: 1) proton density and T2-weighted double turbo spin echo (SE) acquired in transverse planes (time repetition [TR]: 4500 ms, time echo [TE]: 12 ms, time to inversion [TI]: 112 ms, field of view [FOV]: 230 × 172 mm, matrix: 320 × 240, slice thickness: 5 mm, number of slices: 24); 2) fluid-attenuated inversion recovery in the same planes as the SE sequence (TR/TE/TI: 8500/109/2000 ms; FOV: 230 × 168 mm, matrix: 256 × 256, slice thickness: 5 mm, number of slices: 24); 3) T1-weighted 3 D images, with partitions acquired in the sagittal plane, using a modified driven equilibrium Fourier transform [42] sequence (TE/TR/TI: 2.4/7.92/910 ms, flip angle: 15°, 1 mm3 isotropic voxels); and 4) diffusion-weighted volumes were also acquired using SE echo-planar imaging (TE/TR: 89/8500 ms, bandwidth: 2126 Hz/voxel, matrix: 128×128, 80 axial slices, voxel size: 1.8×1.8×1.8 mm) with 30 isotropically distributed orientations for the diffusion sensitizing gradients at a b value of 1000 s/mm2 and 6 b = 0 images. Scanning was repeated 3 times to increase the signal-to-noise ratio.

Images were visually inspected for gross anatomical abnormalities by 2 experienced observers (a neuropsychologist expert in neuroimaging and a neuroradiologist) blind to participant identities.

Images were also visually inspected for movement artifacts, which are a common source of concern while studying HD. Since movement can compromise CC tracking, we excluded subjects who had excessive movement in their scans.

### DTI Processing

Diffusion-weighted images were processed with FMRIB’s Software Library (FSL 4.1 www.fmrib.ox.ac.uk/fsl/). The non-diffusion-weighted images were skull stripped using FSL’s Brain Extraction Tool (BET) (http://www.fmrib.ox.ac.uk/fsl/bet2/index.html), and used to mask all diffusion-weighted images [43]. Images were corrected for eddy current distortion, followed by FSL FLIRT for motion correction. After corrections, the DTI data was averaged and concatenated into 31 (1 B0+30 B1000) volumes. A diffusion tensor model was fitted at each voxel using Diffusion Toolkit, generating FA, AD, and RD maps. RD was defined as the average of the second and third eigenvalues of the diffusion tensor, while AD corresponded to the first eigenvalue.

### Tractography

Tractography methods are outlined in more detail in [37], however, the tractography and ROI drawing was modified to use TrackVis, an interactive environment for fiber tracking reconstruction, display and analysis developed at the Harvard Medical School Martinos Center for Biomedical Imaging at Massachusetts General Hospital (www.trackvis.org). The FACT approach was used to reconstruct fiber paths. For details of the TrackVis tracking algorithm see [44]. A track angle threshold of 35° was used as well as an image mask based on the B0 image to restrict tracking to biologically plausible results.

It is important to note that tractography is still an emerging technology and limitations such as the fact that fiber bundles are not reconstructed directly; instead trajectories are calculated through the data, which are (hopefully) largely parallel to nerve fibers. As such, interpretations of diffusion data should keep in mind that we cannot as of yet assert categorically that scores are being driven by a specific biological or physiological process [14].

Tractography of the CC was performed by manually drawing regions of interest on each individuals FA color map by a single expert (OP) who was blinded to subject age, gender, and diagnosis.

To determine intra-rater reliability, fiber tracts were identified in 10 randomly chosen brain volumes. Reliability was assessed using the intraclass correlation coefficient (2-way mixed for intra-rater). Statistical analyses were performed using SPSS 14.0. Excellent intra reliability was achieved for ROI placement as determined by computing the intra-class correlation coefficients for tract volume and mean FA, AD and RD (Table S1).

Tractography was performed using the two region-of-interest approach. One region was drawn on a midsagittal slice encompassing the entire CC, and seven separate regions of interest spanning both sides of the midline were used as target regions to segment the CC into distinct sections. All regions of interest were drawn according to specific anatomical landmarks and guidelines that were followed carefully and consistently for each individual; the ROI placements are described by [38]. Ordered from front to back the seven sections delineated were: orbital frontal (OF), anterior frontal (AF), superior frontal (SF), superior parietal (SP), posterior parietal (PP), temporal (Temp), and occipital (Occ). ROI locations and exclusion criteria are further detailed in [37]. Fibers that were clearly not part of the anatomical connectivity of the track were eliminated. FA, AD, and RD were calculated for each region by averaging all voxels over the entire tract, counting each voxel only once.

### Statistical Analysis

Demographic differences were assessed using chi-square, independent sample t-tests or Anova as appropriate.

To test for callosal differences, a Multivariate Analysis of Variance (Manova) was applied to all three groups (healthy controls, HD patients and Pre-HD subjects). Sex and age were included as covariates in the model. After that, contrasts were run to individuate the significant difference between groups. To control for multiple comparisons, we used a False Discovery Rate (FDR) threshold p<0.05.

In order to investigate which early callosal changes in the course of pathology were related to clinical, cognitive and genetic aspects, we performed, correlations analysis between CAG repeat length, Disease burden, clinical scales (UHDRS-Motor, -Cognitive), and track measures. Track measures were limited to tract regions and measures already compromised in Pre-HD subjects (main analysis). Operationally we took in consideration only those tract regions and measures that were significantly different between healthy controls and Pre-HD subjects. From those tracts and measures, we extracted values both from the Pre-HD and HD groups. We included these extracted values in the correlational analysis together with the above-mentioned genetic, cognitive and clinical scores. Sex and age were included as covariates. To control for multiple comparisons, we used a False Discovery Rate (FDR) threshold p<0.05.

## Results

### Subject Demographic

Details are outlined in Table 1. Pre-HD subject and HD patients differed in age and CAG repetition length. Additionally, as expected, HD patients had significantly poorer performances with respect to all measures assessed by the UHDRS, and also a significantly higher score of Disease Burden.

### Tractography Data

**Figure 1:**
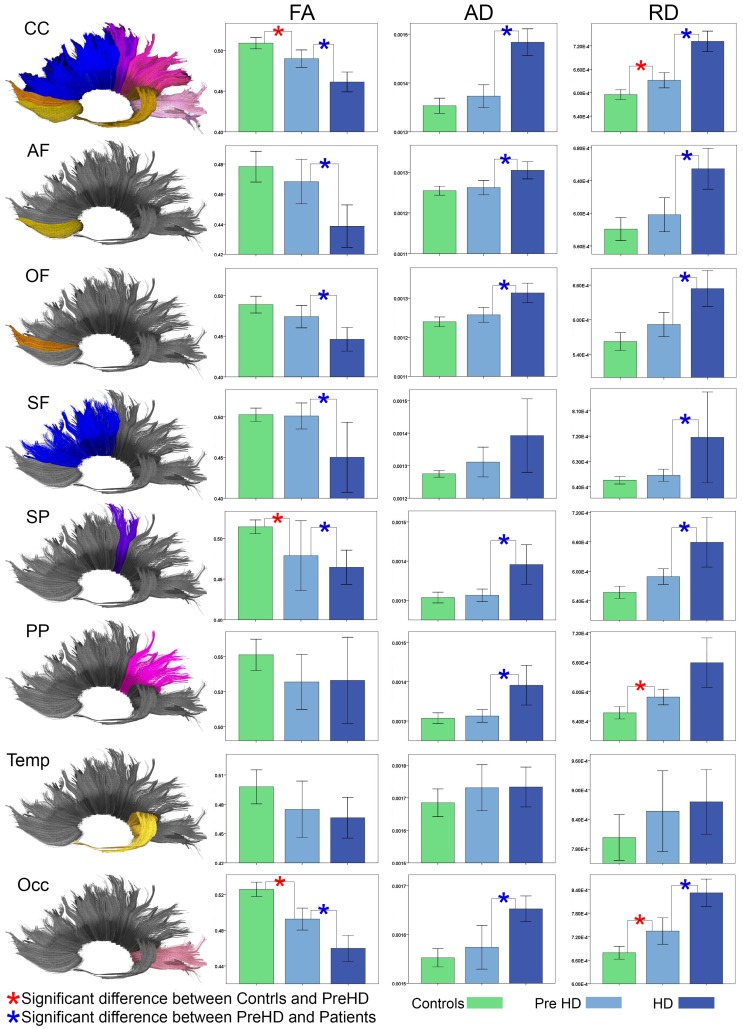
Corpus Callosum Tract Measures by Diagnosis. Bar graphs show differences between CC FA, AD, and RD for the whole CC and the seven components of the CC as defined by the tract target region. The error bars represent the Standard Error Mean (SEM). Tracts: Whole corpus callosum (CC); orbital frontal (OF), anterior frontal (AF), superior frontal (SF), superior parietal (SP), posterior parietal (PP), temporal (Temp), and occipital (Occ).

Statistical details for Manova analysis are provided in Table 2. Track measures are outlined in Table S2. Figure 1 further provides a graphical overview of the findings as well as an indication of significant results.

**Table 2:** Tractography Group Comparisons.

Region		MANOVA			Controls vs Pre-HD	Controls vs HD	Pre-HD vs HD
		F	Df	p	P	p	p
	FA	26.363	2,95	**.001**	**0.007**	**0.001**	**0.001**
Whole CC	AD	50.785	2,95	**.001**	0.356	**0.001**	**0.001**
	RD	67.741	2,95	**.001**	**0.009**	**0.001**	**0.001**
	FA	10.66	2,95	**.001**	0.350	**0.001**	**0.003**
Orbital Frontal	AD	12.00	2,95	**.001**	0.613	**0.001**	**0.001**
	RD	17.07	2,95	**.001**	0.272	**0.001**	**0.005**
	FA	12.28	2,95	**.001**	0.140	**0.001**	**0.005**
Anterior Frontal	AD	19.85	2,95	**.001**	0.230	**0.001**	**0.001**
	RD	20.51	2,95	**.001**	0.080	**0.001**	**0.001**
	FA	7.917	2,92	**.001**	0.984	**0.001**	**0.002**
Superior Frontal	AD	5.71	2,92	**.001**	0.323	**0.001**	0.043
	RD	5.91	2,92	**.001**	0.774	**0.001**	**0.010**
	FA	15.67	2,91	**.001**	**0.013**	**0.001**	**0.007**
Superior Parietal	AD	13.70	2,91	**.001**	0.795	**0.001**	**0.001**
	RD	20.41	2,91	**.001**	0.034	**0.001**	**0.001**
	FA	1.84	2,94	.184	–	–	–
Posterior Parietal	AD	8.63	2,94	**.001**	0.120	**0.001**	**0.023**
	RD	7.75	2,94	**.001**	**0.02**	**0.001**	0.203
	FA	2.56	2,95	.082	–	–	–
Temporal	AD	1.14	2,95	.325	–	–	–
	RD	1.72	2,95	.186	–	–	–
	FA	40.87	2,95	**.001**	**0.001**	**0.001**	**0.001**
Occipital	AD	14.32	2,95	**.001**	0.382	**0.001**	**0.001**
	RD	39.90	2,95	**.001**	**0.006**	**0.001**	**0.001**

HD = Huntington’s disease; Pre-HD = gene-positive, without motor symptoms.

**In bold:** significant results after correction for multiple comparisons (FDR p<0.05).

#### FA

Callosal FA analysis revealed a number of significant findings between healthy controls and Pre-HD subjects. Within the CC as a whole FA was lower in Pre-HD subjects as well as within the tracts connecting to the SP, and Occ regions. In FA comparisons between healthy controls and HD patients, reduced FA in patients was seen across the whole CC and within the tracts connecting to the OF, AF, SF, SP, and Occ regions. Finally, FA analyses between Pre-HD subjects and HD patients revealed reductions in HD patients across the whole CC and within the tracts connecting the OF, AF, SF, SP, and Occ regions.

#### AD

HD patients exhibited higher AD compared to healthy controls across the whole CC and within the tracts connecting the OF, AF, SF, SP, PP, and Occ regions. The analyses between Pre-HD subjects and HD patients indicated higher AD in HD patients across the CC as a whole and within the tracts connecting the OF, AF, SP, PP and Occ regions.

#### RD

RD analyses showed significant increases in Pre-HD subjects compared to healthy controls across the CC as a whole as well as within the tracts connecting the PP, and Occ regions. HD patients also showed increased RD compared to healthy controls across the CC as a whole and tracts within the OF, AF, SF, SP, PP and Occ regions. Finally, comparisons between Pre-HD subjects and HD patients showed increased RD in HD patients across the CC as a whole and within the tracts connecting the OF, AF, SF, SP, and Occ regions.

### Correlations

**Figure 2:**
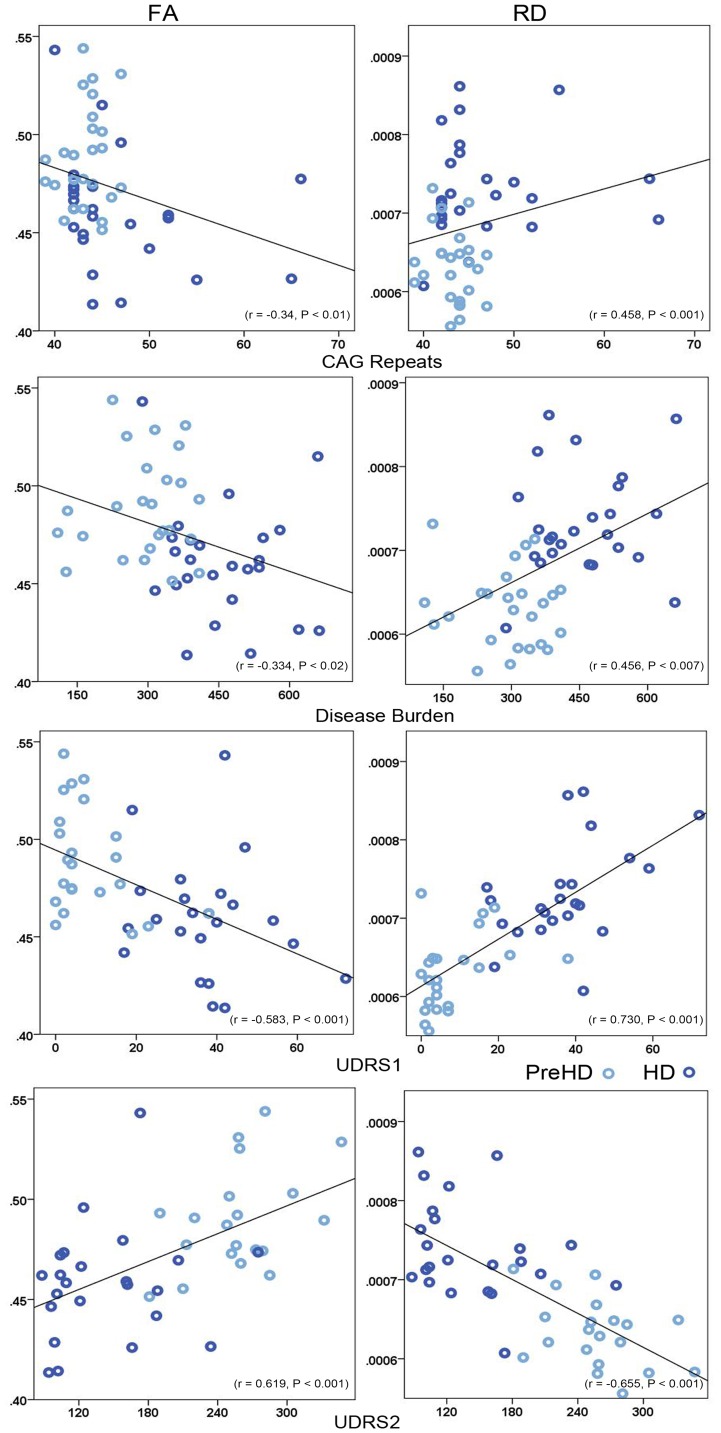
Significant Correlations between Corpus Callosum FA, RD and CAG Repeat Length, Disease Burden, UDRS1 & UDRS2.

Correlations between callosal measures, clinical scores and genetic value within Pre-HD subjects and HD patients were significant for CAG Repeat length, Disease Burden, UHDRSI, and UHDRS2. Scatterplots in Figure 2 display correlations between the whole CC FA and RD and CAG repeats, Disease Burden, UHDRSI and UHDRS2.

CAG Repeat length was negatively correlated with whole CC FA (*r* = –0.34, *p*<0.01), and Occ FA (*r* = –0.363, *p*<0.011). Positive correlations were found between the whole CC RD (*r = *0.458, *p*<0.001) and Occ RD (*r* = 0.358, *p*<0.012).

Disease Burden was negatively correlated with FA of the whole CC (*r* = –0.334, *p*<0.02) and Occ (*r* = –0.319, *p*<0.027). Positive correlations with RD across the CC as a whole (*r* = 0.456, *p*<0.007) and the Occ (*r* = 0.323, *p*<0.025) were found.

UHDRS1 was negatively correlated with whole CC FA *(r* = –0.583, *p*<0.001), SP FA (*r* = –0.492, *p*<0.002) and Occ FA (*r* = –0.515, *p*<0.001). UHDRS1 was positively correlated with RD with the CC as a whole (*r* = 0.730, *p*<0.001) and Occ component (*r* = 0.559, *p*<0.001). UHDRS2 was positively correlated with whole CC FA (*r* = 0.619, *p*<0.001), and Occ FA (*r* = 0.481, *p*<0.001). UHDRS2 was negatively correlated with whole CC RD (*r* = –0.655, *p*<0.001) and Occ RD (*r* = –0.452, *p*<0.003).

## Discussion

This study adds four main findings to the HD literature. One, greater RD in Pre-HD subjects compared to healthy controls, adds support to the hypothesis that demyelination occurs prior to disease onset in the CC revealing abnormal structural connectivity. Two, increased RD appears to occur earlier in occipital and posterior parietal tracks compared to other regions. These tracts are associated with motor and visual systems early involved in HD. Three, structural connectivity of the CC is correlated with motor and cognitive scores in patients (UHDRS1 and UHDRS2 scores), which indicates that the fibers play an important functional role. Further, this indicates that the diffusivity differences between healthy controls and patients reflect connectivity impairments both at structural and functional level. Four, increased Disease Burden and CAG repeat length is associated with altered CC microstructure suggesting a strong genetic basis for WM impairments.

Using a validated ROI approach for identifying the CC and its major sub regions [37], [38], we were able to examine highly localized changes of WM connectivity in the CC. We used FA, AD and RD to assess track structure. Significant differences were found between Pre-HD subjects and healthy controls for FA and RD. This result is similar to a recent study which used a related method [25], and found increased apparent diffusion coefficient (ADC) across the whole CC tract, though they did not find FA differences and did not examine AD/RD. Our finding of decreased FA may have to do with varying acquisition strategies or subject demographics. As axial and radial measures make up the FA value, significant RD increases but not significant AD increases indicate myelin impairment in Pre-HD stages may be the driving factor in decreased FA. Three previous studies using varying DTI methods found similar RD increases in Pre-HD subjects compared to healthy controls [23], [24], [45]. Our result of increased RD supports the previous research. Further evidence for impaired myelin in HD comes from a DTI study on a HD mouse model [46], which found thinner myelin sheaths and increased myelin periodicity.

When comparing Pre-HD subjects and HD patients, results indicate significant deterioration with disease progression characterized by both increased AD and RD across the whole CC. This suggests that the CC may be susceptible to both axonal damage and demyelination as the disease progresses. Previous studies have also revealed the negative effect HD has on the CC [21], [23], [24], [25].

However, although it is likely HD progression results in increased demyelination, we are not yet able to discern whether abnormal brain development in Pre-HD subjects contributed to their reduced myelination or whether disease progression results in demyelinated fibers. Previous work has suggested that abnormal brain development may contribute to the pathogenesis of HD as a precursor to the neurodegenerative process. Also, it may be some combination of abnormal brain development and pathogenesis that is contributing to the connectivity deficits [47]. Future longitudinal studies on Pre-HD subjects will be needed to elucidate this hypothesis.

Regional analysis of the CC revealed increases in RD in the tracts connecting parietal and occipital regions in Pre-HD subjects compared to healthy controls. It is likely the functions of the tracts are closely tied to the regions they connect with and both the motor and visual systems show early impairment in HD. For example, cortical thickness is reduced in occipital and parietal lobe regions in Pre-HD subjects [48] and cortical thickness has been shown to correlate with connectivity properties of WM tracts [29], [49]. Moreover, decreased FA was seen in the SP and Occ tracts, which, taken together support a possible posterior to anterior gradient for disease progression [23], [50], [51]. Finally, it has also been suggested [23] that metabolic dysfunction and alterations in energetics [52] seen in HD may influence the CC. Our regionally specific results of early changes in the Occ, PP, SP tracts add possible support to this hypothesis as these tracts are associated with motor and visual systems and are therefore subject to high energetic demands.

In the current study, we also found correlations between global motor & cognitive (UHDRS1 & UHDRS2) scores and CC connectivity. This relationship was observed across the CC as a whole and within specific sub regions (SP, and Occ) for FA and RD. This suggests that the CC may play an important functional role in HD symptoms. Furthermore, because diffusivity is associated with decreased motor and cognitive abilities, it supports the conclusion that WM diffusion differences between patients and healthy controls reflects abnormal structural and eventually functional connectivity between cortical regions connected by the CC. The correlations with RD indicate that decreased fiber myelination likely results in reduced global motor and cognitive scores. An earlier paper on HD patients also found a correlation between motor scores and the body of the CC using MD/RD [22] that was localized to premotor and supplementary motor cortices. The possible important role for the CC in clinical symptoms of HD is further supported by the significant relationship between cognitive measures and reductions in FA and increased RD shown by Rosas et al. [23].

However the full picture can be more complex. As CC connectivity is correlated with Disease Burden as well, which is a measure of disease severity, it is possible that connectivity deficits in the CC do not play a particularly focal role in motor and cognitive changes associated with HD. Rather, the CC may play a more general role in explaining the cognitive symptoms of HD. Importantly though, as reported previously [23], the cognitive symptoms of HD have not been adequately explained by cortico-striatal dysfunction alone. This indicates a possible district role for the CC and in general of WM in HD symptoms.

We also investigated the role CAG repeats may play in CC connectivity. We found a significant (though weak) correlation between CAG repeat length and connectivity across the CC as a whole that was driven by increased RD. This finding indicates less myelination in subjects with higher CAG repeats. However, Disease Burden showed a stronger correlation with CC connectivity, indicating that CAG repeats have an increasingly negative effect on the CC as the subject ages. This genetic basis for WM alterations was not found in two previous diffusion CC studies [22], [23], where CAG repeat length did not correlate with diffusivity measures. Again, this may have to do with the increased sensitivity of tractography to elucidate the microstructural changes in WM or varying subject demographics.

In conclusion, the CC is made up of thousands of WM fibers and we have shown, using a DTI-tractography method, a regional callosal variation in diffusivity measures between Pre-HD subjects and healthy control subjects. These structural connectivity impairments are present before a subject is clinically defined as having HD. Impairments appear to start in motor and visual tracts and proceed to encompass most of the CC in a posterior to anterior direction. And although the biological role of this connectivity change is still not fully understood [14], and caution should be used in the interpretation of diffusion findings [53], it is likely demyelination occurs early and later includes axonal damage with disease progression. Furthermore, abnormal CC structural connectivity is correlated with motor and cognitive scores (UHDRS1 & 2), suggesting an important functional role for these callosal tracts.

## Supporting Information

Table S1 **Intra-rater Reliablity Coefficient.**
(DOC)

Table S2 **Tractography Data.**
(DOC)
