# Supplementary material for: Tractography of the Corpus Callosum in Huntington’s Disease
Source: PLoS One. 2013 Sep 3;8(9):e73280. doi: 10.1371/journal.pone.0073280 (PMC3760905; doi:10.1371/journal.pone.0073280)
Supplement: Table S1 — Intra-rater Reliablity Coefficient. (DOC) [file pone.0073280.s001.doc]

|  | **Intra-rater Reliability Coefficient** | | | |
| --- | --- | --- | --- | --- |
|  | **Mean Volume** | **Mean FA** | **Mean AD** | **Mean RD** |
| **Region** |  |  |  |  |
| Whole CC | .995 | .989 | .969 | .974 |
| OF | .959 | .993 | .966 | .985 |
| AF | .963 | .981 | .957 | .946 |
| SF | .927 | .923 | .953 | .888 |
| SP | .917 | .934 | .942 | .911 |
| PP | .998 | .997 | .989 | .997 |
| Temp | .918 | .935 | .935 | .945 |
| Occ | .944 | .979 | .990 | .989 |

**Table S1.** Intra-rater Reliablity Coefficient

CC = corpus callosum; OF = Orbital Frontal; AF = Anterior Frontal; SF = Superior Frontal; SP: Superior Parietal; PP = Posterior Parietal; Temp = Temporal; Occ = Occipital; FA = fractional anisotropy; AD = axial diffusivity; RD = radial diffusivity.
