# Supplementary material for: Tractography of the Corpus Callosum in Huntington’s Disease
Source: PLoS One. 2013 Sep 3;8(9):e73280. doi: 10.1371/journal.pone.0073280 (PMC3760905; doi:10.1371/journal.pone.0073280)
Supplement: Table S2 — Tractography Data. (DOC) [file pone.0073280.s002.doc]

**Table S2.** Tractography Data

|  | **FA (mean ± SD)** | | | **AD (mean ± SD)** | | | **RD (mean ± SD)** | | |
| --- | --- | --- | --- | --- | --- | --- | --- | --- | --- |
| **Region** | Controls | Pre-HD | HD | Controls | Pre-HD | HD | Controls | Pre-HD | HD |
| Whole CC | .508  ±.027 | .496  ±.027 | .454  ±.03 | 1.35E-03 ±4.60E-05 | 1.35E-03 ±6.50E-05 | 1.50E-03 ±9.10E-05 | 6.00E-04 ±4.50E-05 | 6.10E-04 ±5.50E-05 | 7.50E-04 ±8.00E-05 |
| Orbital Frontal | .478  ±.039 | .478  ±.041 | .429  ±.039 | 1.25E-03 ±4.10E-05 | 1.26E-03 ±4.40E-05 | 1.31E-03 ±5.30E-05 | 5.80E-04 ±5.60E-05 | 5.80E-04 ±5.90E-05 | 6.70E-04 ±6.80E-05 |
| Anterior Frontal | .488  ±.040 | .484  ±.036 | .437  ±.040 | 1.24E-03 ±3.90E-05 | 1.25E-03 ±4.90E-05 | 1.32E-03 ±7.40E-05 | 5.60E-04 ±5.80E-05 | 5.70E-04 ±5.70E-05 | 6.70E-04 ±9.20E-05 |
| Superior Frontal | .502  ±.030 | .504  ±.038 | .394  ±.173 | 1.28E-03 ±3.90E-05 | 1.31E-03 ±1.11E-04 | 1.23E-03 ±5.20E-04 | 5.60E-04 ±4.90E-05 | 5.80E-04 ±5.00E-05 | 6.30E-04 ±4.16E-04 |
| Superior Parietal | .514  ±.029 | .479  ±.10 | .316  ±.223 | 1.31E-03 ±4.80E-05 | 1.25E-03 ±2.63E-04 | 9.50E-04 ±6.71E-04 | 5.60E-04 ±4.40E-05 | 5.60E-04 ±1.22E-04 | 4.50E-04 ±3.30E-04 |
| Posterior Parietal | .551  ±.041 | .535  ±.046 | .404  ±.238 | 1.32E-03 ±6.40E-05 | 1.35E-03 ±9.90E-05 | 1.10E-03 ±6.47E-04 | 5.10E-04 ±5.10E-05 | 5.40E-04± 6.30E-05 | 4.50E-04 ±2.82E-04 |
| Temporal | .468  ±.132 | .479  ±.071 | .462  ±.051 | 1.58E-03 ±4.29E-04 | 1.72E-03 ±1.76E-04 | 1.74E-03 ±1.50E-04 | 7.60E-04 ±2.48E-04 | 8.50E-04± 2.04E-04 | 8.90E-04 ±1.60E-04 |
| Occipital | .526  ±.029 | .498  ±.030 | .455  ±.035 | 1.55E-03 ±6.70E-05 | 1.56E-03 ±1.15E-04 | 1.67E-03 ±7.10E-05 | 6.80E-04 ±6.00E-05 | 7.20E-04 ±8.90E-05 | 8.50E-04 ±8.70E-05 |

FA = fractional anisotropy; AD = axial diffusivity; RD = radial diffusivity; CC = corpus callosum; HD = Huntington’s disease; Pre-HD = gene-positive, without motor symptoms.
